# NeuralEE: A GPU-Accelerated Elastic Embedding Dimensionality Reduction Method for Visualizing Large-Scale scRNA-Seq Data

**DOI:** 10.3389/fgene.2020.00786

**Published:** 2020-10-06

**Authors:** Jiankang Xiong, Fuzhou Gong, Lin Wan, Liang Ma

**Affiliations:** ^1^National Center for Mathematics and Interdisciplinary Sciences, Academy of Mathematics and Systems Science, Chinese Academy of Sciences (CAS), Beijing, China; ^2^School of Mathematical Sciences, University of Chinese Academy of Sciences, Beijing, China; ^3^Key Laboratory of Zoological Systematics and Evolution, Institute of Zoology, Chinese Academy of Sciences (CAS), Beijing, China

**Keywords:** single-cell RNA sequencing, elastic embedding, neural networks, large-scale, stochastic optimization, parametric models, generalizable models

## Abstract

The dramatic increase in amount and size of single-cell RNA sequencing data calls for more efficient and scalable dimensional reduction and visualization tools. Here, we design a GPU-accelerated method, NeuralEE, which aggregates the advantages of elastic embedding and neural network. We show that NeuralEE is both scalable and generalizable in dimensional reduction and visualization of large-scale scRNA-seq data. In addition, the GPU-based implementation of NeuralEE makes it applicable to limited computational resources while maintains high performance, as it takes only half an hour to visualize 1.3 million mice brain cells, and NeuralEE has generalizability for integrating newly generated data.

## 1. Introduction

Dimensionality reduction is one of the basic steps in machine learning algorithms and big-data analyses, especially in the analysis of high-throughput single cell RNA sequencing data (scRNA-seq data). scRNA-seq enables us to simultaneously profile thousands of genetic markers at single-cell resolution, which makes it an ideal tool to study the cell-cell heterogeneity in developmental biology, oncology, and immunology. Visualization of scRNA-seq data in a manageable dimension often plays as a pivotal first step prior to other downstream analyses such as cell type identification or cell developmental trajectory reconstruction.

Among the numerous dimensionality reduction and visualization methods, t-distributed stochastic neighbor embedding (t-SNE) (van der Maaten and Hinton, 2008) is most widely used in the single-cell community to visualize data structures. While t-SNE emphasizes the neighborhood information, which keeps the local affinity of the data, it tends to shatter the global structures (Becht et al., 2018). As an extension of stochastic neighbor embedding (SNE), elastic embedding (EE) algorithm penalizes, placing far apart latent points from similar data points and placing close together latent points from dissimilar data points (Carreira-Perpinán, 2010), thereby preserving the intrinsic data structure both locally and globally (Hie et al., 2020). EE has been recently proved to be well-performed in visualization and in reconstruction of the embedded structure of the cell developmental process (An et al., 2019; Chen et al., 2019).

Recent advances in automatic cell isolation and multiplex sequencing have led to an exponential growth in the number of cells (may reach the order of millions) sequenced for individual studies (Svensson et al., 2018). Many modified version of dimensionality reduction and visualization methods, such as net-SNE (Cho et al., 2018), FIt-SNE (Linderman et al., 2019), and UMAP (Becht et al., 2018), have been introduced to deal with data in large scale. Net-SNE in particular optimizes the original t-SNE under a neural network (NN) framework. In addition, methods based on autoencode, which is in form of a specialized NN framework, have been proposed to deal with scRNA-seq data for dimensionality reduction. To list a few, scScope (Deng et al., 2019) reconstructs scRNA-seq data by a deep recurrent autoencoder. DCA (Eraslan et al., 2019) models scRNA-seq for count data by a deep count autoencoder. scVI (Lopez et al., 2018), based on a variational autoencoder (Kingma and Welling, 2014), also models count data and incorporates batch correction in addition to dimensionality reduction. Many other methods are also constructed under the deep learning framework (Ding et al., 2018; Wang and Gu, 2018) that take advantage of parallel and scalable features in deep neural networks (Lin et al., 2020). EE optimization procedures still lack sufficient scalability to mega-scale datasets.

Here, we develop neural elastic embedding termed NeuralEE, a scalable and generalizable method that trains a NN with a mini-batch trick, mapping from high-dimensional single-cell gene-expression profiles to a low-dimensional visualization. NeuralEE visualizes large-scale scRNA-seq data very efficiently and accurately on a conventional workstation with GPU-installed, making it applicable to biological labs with limited computational resources. We also validate the accuracies of visualization by NeuralEE on four benchmark datasets and a simulated dataset. Furthermore, NeuralEE has no computational restrictions on embedding dimensions, making it viable as a general purpose dimension reduction technique.

## 2. Materials and Methods

### 2.1. EE

Given *Y*_*D*×*N*_ = (*y*_1_, …, *y*_*N*_) the *D* × *N* matrix of the scRNA-seq dataset with *N* cells in ℝ^*D*^, where *D* is typically on the order of tens of thousands (number of genes), EE seeks to find the low-dimensional embedding on ℝ^*d*^, *X*_*d*×*N*_ = (*x*_1_, …, *x*_*N*_), with *d*≪*D*. Formally, EE solves the following optimization problem:

(1) $$\begin{array}{l} \underset{X}{\min}E\left(X\right)=\underset{X}{\min}\underset{n\neq m}{\sum }w_{nm}^{+}{\left\|x_{n}-x_{m}\right\|}^{2}\\ +\lambda \underset{n\neq m}{\sum }w_{nm}^{-}\exp\left(-{\left\|x_{n}-x_{m}\right\|}^{2}\right), \end{array}$$

where the attractive weights $W_{N\times N}^{+}=\left(w_{nm}^{+}\right)$ and the repulsive weights $W_{N\times N}^{-}=\left(w_{nm}^{-}\right)$ are both *N* × *N* symmetric and non-negative matrices, which are derived from *Y*. The parameter λ trades off between the attractive and repulsive terms. The *W*^+^ can be defined as Gaussian affinities (Hinton and Roweis, 2003) or entropic affinities (Vladymyrov and Carreira-Perpinan, 2013), and the *W*^−^ can be simply defined as Euclidean distance. The objective function *E*(*X*) is normally solved by fixed-point iteration (Carreira-Perpinán, 2010) or partial-Hessian strategies (Vladymyrov and Carreira-Perpinan, 2012). However, when *N* is large, the optimization can be computationally expensive.

### 2.2. NeuralEE

The original EE is not generalizable: it is unable to project new samples to existing embedding. A basic approach is to use a mapping **F** belonging to a parametric family F of mappings, then involve **F** in the learning from the beginning, by replacing *x*_*n*_ with **F**(*y*_*n*_) in the embedding objective function and optimizing it over the parameters of **F**.

An NN with sufficient hidden layers (with non-linear activation functions) is capable of approximate functions with arbitrary complexity (Leshno et al., 1991). We can thus choose to use a standard feedforward NN as the parametric family F of mappings for EE. Standard feedforward NN architecture is a multilayer stack of simple modules that maps a fixed-size input (for example, a cell represented with *D* genes expression) to a fixed-size output (for example, coordinate in embedded space). To go from one layer to the next, a set of units (neurons) compute weighted sums of inputs from their previous layer and pass the results to the next layer through a non-linear function. Units that are not in the input (the first) or output (the last) layers are conventionally called hidden units. The hidden layers can be seen as distorting the input in a non-linear way so that coordinates in embedded space become linearly separable by the last layer. Multilayer architectures can also be trained by simple stochastic gradient descent. As long as the modules are relatively smooth functions of their inputs and of their internal weights, one can compute gradients using the backpropagation procedure (Lecun et al., 2015).

We propose NeuralEE, which applies the NN framework with mini-batch trick to mitigate the high computational intensity when dealing with large-scale datasets. NeuralEE defines a NN that maps data from the original space to the embedded space. The embedding represents as the function of the original data is fed into Equation (1) with attractive weight and the repulsive weight matrices which are calculated offline. By use of the backpropagation algorithm (Lecun et al., 1990), the parameters in the NN are optimized, and the mapping of the embedding is thus learned.

In detail, with arbitrary parametric mapping $Net_{\theta }:R^{D}\to R^{d}$ defined by NN, the objective *E*(*X*) in Equation (1) can be written as *E*(*Net*_θ_(*Y*)), where *Net*_θ_(*Y*) = (*Net*_θ_(*y*_1_), …, *Net*_θ_(*y*_*N*_)) (the specific structure of NN is detailed at Supplementary Materials).

The parameters θ can thus be optimized via gradient descent algorithm by applying the chain rule as follows:

(2) $$\begin{array}{l} \frac{\partial E\left(Net_{\theta }\right)}{\partial \theta }=\overset{N}{\underset{n=1}{\sum }}{\left(\frac{\partial E\left(X\right)}{\partial x_{n}}\right)}^{T}\frac{\partial Net_{\theta }\left(y_{n}\right)}{\partial \theta }, \end{array}$$

where $\frac{\partial E\left(X\right)}{\partial x_{n}}$ has close-form expression.

(3) $$\begin{array}{l} \frac{\partial E\left(X\right)}{\partial x_{n}}=4\underset{m\neq n}{\sum }\left(w_{nm}^{+}-\lambda w_{nm}^{-}\exp\left(-{\left\|x_{n}-x_{m}\right\|}^{2}\right)\right)\left(x_{n}-x_{m}\right). \end{array}$$

And $\frac{\partial Net_{\theta }\left(y_{n}\right)}{\partial \theta }$ can be acquired by backpropagation algorithm.

### 2.3. Stochastic Optimization

**Figure 1:**
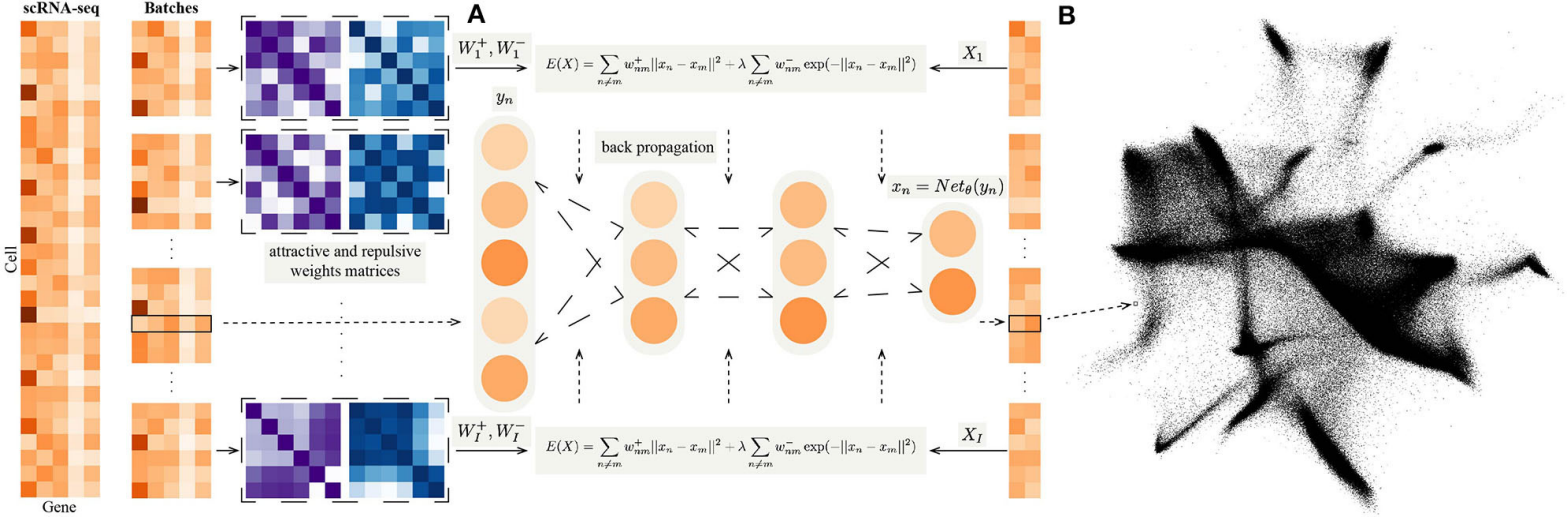
**(A)** The flow chart of NeuralEE. In brief, NeuralEE constructs an NN that defines a parametric mapping from the original space to the embedded space. The full dataset is first randomly partitioned into several batches (only one batch is also acceptable, which means not applied with mini-batch trick or stochastic optimization). On each batch the attractive weight and the repulsive weight matrices are calculated and fed into the loss function of EE, which is represented as a composite function of the original data. By backpropagation algorithm, the parameters in the NN are optimized, and the mapping of the embedding is thus learned. **(B)** Visualization results of 1.3 million mouse brain cells by NeuralEE-SO.

In cases where *N* is large, the calculation of $\frac{\partial E\left(X\right)}{\partial x_{n}}$ can be time-consuming. Additionally, it will be memory-costly to store the attractive and the repulsive weight matrices. We therefore further propose a stochastic optimization version of NeuralEE, termed NeuralEE-SO, by applying the mini-batch trick. It first randomly partitions the full matrix into several batches *B* = {*b*_*i*_ ⊂ {1, …, *N*}} before it then calculates the attractive weight and the repulsive weight matrices for each batch. At each iteration of backpropagation, the gradients of NN parameters are calculated on each batch and averaged over all batches:

(4) $$\begin{array}{l} \frac{\partial E\left(Net_{\theta }\right)}{\partial \theta }\approx \gamma \frac{1}{\left|B\right|}\overset{\left|B\right|}{\underset{i=1}{\sum }}\underset{n\in b_{i}}{\sum }{\left(\frac{\partial E\left(X_{i}\right)}{\partial x_{n}}\right)}^{T}\frac{\partial Net_{\theta }\left(y_{n}\right)}{\partial \theta }, \end{array}$$

where γ is an offset constant, which is finally integrated into the learning rate of the gradient descent algorithm. The flowchart of NeuralEE is shown at Figure 1A, and we give the pseudocode of NeuralEE at Supplementary Algorithm 1.

### 2.4. Data

We test NeuralEE on five scRNA-seq datasets, including 3,005 mouse cortex cells (Zeisel et al., 2015) (hereinafter denoted as **CORTEX**), 4,016 hematopoietic stem and progenitor cells. Tusi et al. (2018) (hereinafter denoted as **HEMATO**), 12,039 human peripheral blood mononuclear cells (Zheng et al., 2017) (hereinafter denoted as **PBMC**), 27,499 mouse retinal bipolar neuron cells (Shekhar et al., 2016) (hereinafter denoted as **RETINA**), 1.3 million mouse brain cells (10x Genomics, 2017) (hereinafter denoted as **BRAIN-LARGE**), and also a simulated complex trajectory of embedded data from Moon et al. (2019) (hereinafter denoted as **ArtificialTree**) (data information is detailed at Supplementary Materials). The pre-filtering of cells and the labeling of cells follow the procedures in Lopez et al. (2018) for the first four data. We further filter and normalize the genes in following steps. First we apply log(1+*x*) transformation to each element of the cell-gene expression matrix. Then we exclude genes with low expression variance, and in most datasets retain the top 500 genes are ordered by variance (558 genes for **CORTEX**). Finally, we normalize the expression of each gene by subtracting its mean and dividing its standard deviation. We preprocess **BRAIN-LARGE** data following the procedures in Zheng et al. (2017) and, as Cho et al. (2018) does, retain the top 50 principal components (PCs) as features. For **ArtificialTree** data, we take the raw data as input.

### 2.5. Quantitative Evaluation

We quantitatively evaluate NeuralEE with other methods on simulation data by comparing their generalization error measured on K-nearest neighbor classifier. First, we apply different dimensionality reduction methods on the entire **ArtificialTree** dataset, where the ground-truth labels of the cells are known. We then conduct a 10-fold cross-validation procedure on the full dataset, and the cross-validation errors for different settings of *K* (1 ≤ *K* ≤ 40) are calculated for each dimensional reduction methods. The minimum cross validation errors of each methods are chosen as the corresponding generalization errors. Selected hyperparameters of NeuralEE and other methods are listed at Supplementary Table 2.

### 2.6. Implementation

NeuralEE is implemented in Python. It integrates the EE optimization protocol of Vladymyrov and Carreira-Perpinan (2012) and the NN framework based on the PyTorch (Paszke et al., 2019) module, which exploits parallel computation of GPU to accelerate the optimization. We provide freely available codes and detailed guidance on our Github site https://github.com/HiBearME/NeuralEE/tree/v0.1.6. In this study, NeuralEE is performed on a 64G computer memory workstation, with a NVIDIA GPU (GCeForce GTX 1080 Ti, 11G video RAM).

## 3. Results

### 3.1. NeuralEE Preserves the Properties of EE

**Figure 2:**
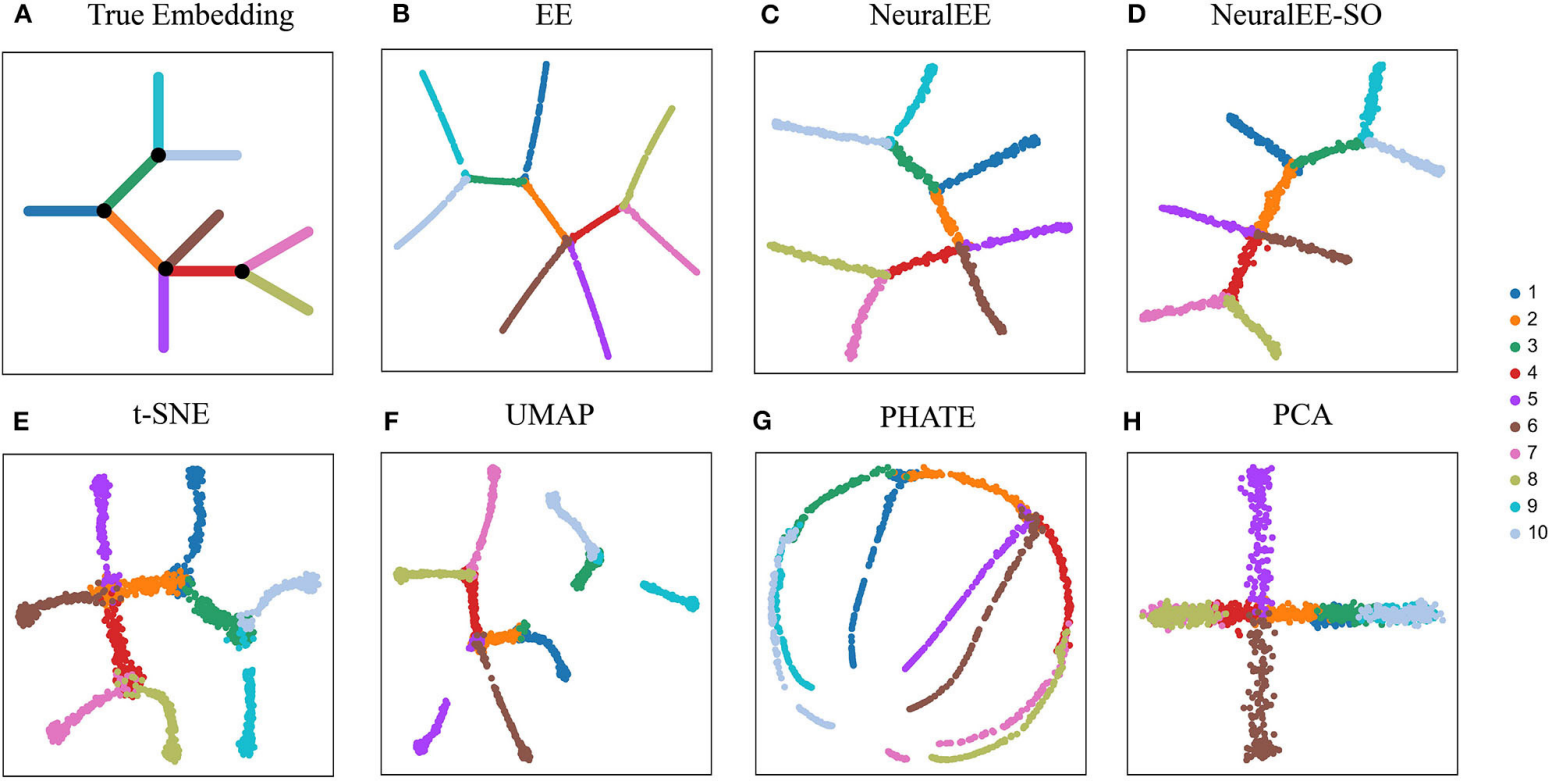
Comparison of NeuralEE to other visualization methods on **ArtificialTree** data. **(A)** True embedding. **(B)** EE. **(C)** NeuralEE. **(D)** NeuralEE-SO. **(E)** t-SNE. **(F)** UMAP. **(G)** PHATE. **(H)** PCA. Different color corresponds different branch of artificial tree.

We first compare NeuralEE to EE and other common dimension reduction methods on **ArtificialTree** data, which contains 1,440 single cells and 60 genes. The **ArtificialTree** data have an embedded continuous tree structure with 10 branches. Each branch constitute of about 140 cells that have exclusive expression on a subset of genes, where the progression along the branch is modeled by gradually increase the expression of these genes (Figure 2A). The two versions of NeuralEEs together with EE resemble each other in results with EE results being more neatly presented (Figures 2B–D). NeuralEE inherits the merits of EE, which can preserve both global and local structure of data. The state-of-the-art methods, t-SNE and UMAP, are also good at keeping the local affinity; however, compared to other methods, they both tend to shatter the global structures (Figures 2E,F). PHATE (Moon et al., 2019) tends to stretch the branches, while PCA fails to resolve all the branches (Figures 2G,H). We further quantitatively compare the performance of NeuralEE to other methods on these simulation data. By performing the K-nearest neighbors classifiers on each embedded space, and with *K* varying from 1 to 40, we calculate their minimum generalization error (Supplementary Table 1). Except for PCA, the minimum generalization errors for other methods are all less than 0.1. NeuralEE, NeuralEE-SO, and EE exhibited the best performance with NeuralEE and EE ties at the top.

We also apply NeuralEE together with other dimension reduction methods on real biological data (Supplementary Figure 1). As expected, both versions of NeuralEE give very similar visualization structures on these datasets as EE. However, when the size of dataset is large, EE suffers from computation problems. For instance, it fails to run the **RETINA** dataset. NeuralEE, and especially NeuralEE-SO, on the other hand, overcome this issue and are able to carry forward the characteristics of EE on larger datasets. The parameter λ in EE (and thus in NeuralEE) trades off between the local affinity and the global structure (Carreira-Perpinán, 2010; Hie et al., 2020). Normally, the results of EE are relatively robust with the setting of λ within a certain range (Carreira-Perpinán, 2010; An et al., 2019; Chen et al., 2019). We use 1 as the default setting of λ in this study; however, there should be little influence in the resulting visualization with λ ranging from 1 to 10 (Supplementary Figure 2). We have used the top 500 variable genes in the above visualizations of real datasets. We also demonstrate that when enough variation retained, rising the number of initializing gene do not alter the main structure of the visualization (Supplementary Figure 3). In practice, one may also choose to use another dimensional reduction method to extract the main features in advance to visualization (Lin et al., 2020; Wu and Zhang, 2020). The dimensionality reduction method can be in linear form such as PCA (Kobak and Berens, 2019) or in non-linear form such as scVI (Lopez et al., 2018; Wu and Zhang, 2020). We compare the visualization performance of NeuralEE with FIt-SNE and UMAP based on 50 PCs or latent variables learned by scVI, and we show that NeuralEE still preserves global structure better than t-SNE and UMAP (Supplementary Figure 4).

### 3.2. NeuralEE Is Generalizable to Newly Generated Data

**Figure 3:**
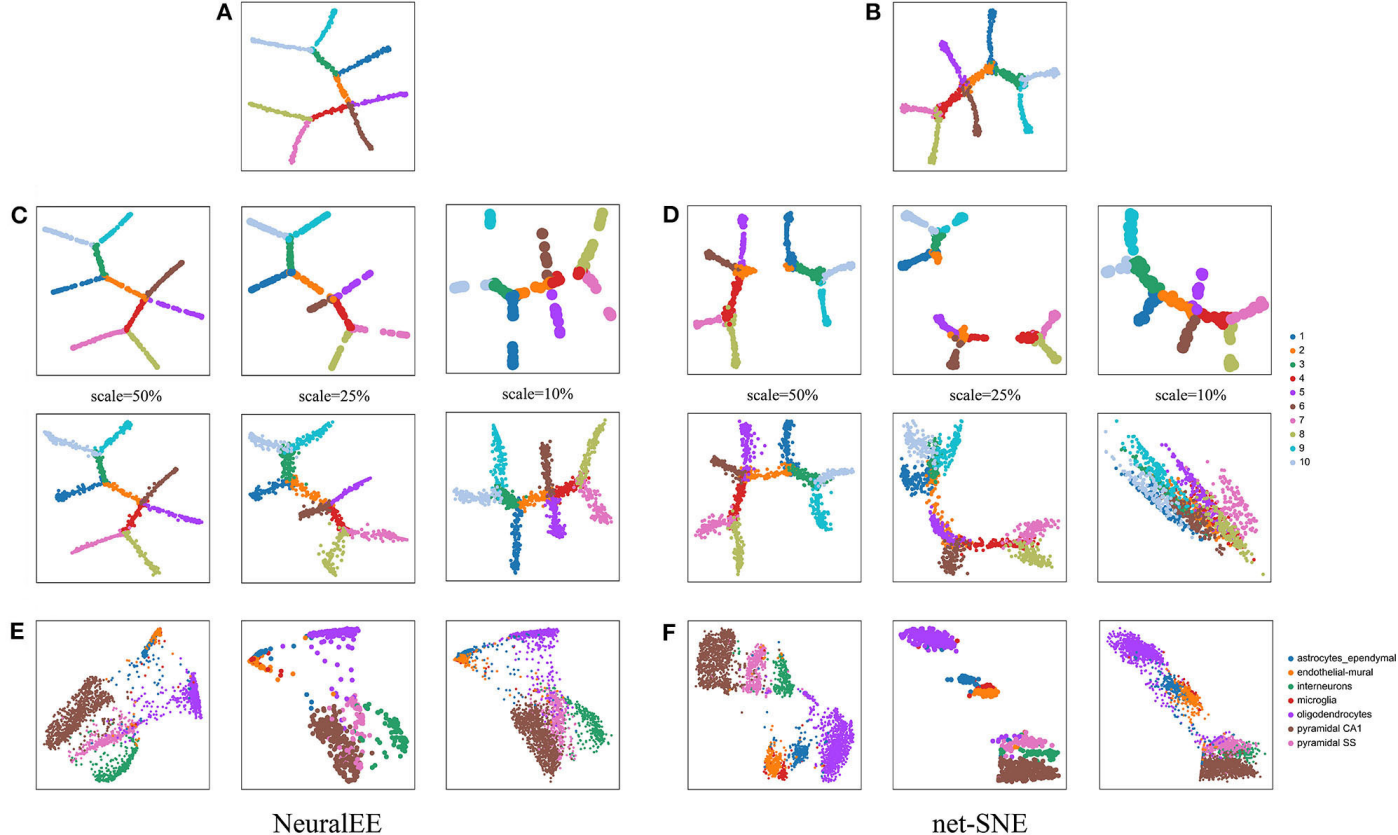
**(A)** NeuralEE or **(B)** net-SNE on the full **ArtificialTree** data. Their embeddings with stochastic optimization are showed at Supplementary Figure 6. **(C)** The top line panel is the NeuralEE based on the sub-samples with a sub-sampling scale as the index *scale*, and the bottom line panel is the mapping of all samples to the embedded space based on the NN trained on the top panel. **(D)** net-SNE under the similar experiments as **(C)**. **(E)** From left to right is the NeuralEE on the entire **CORTEX** data, NeuralEE based on the sub-samples with a sub-sampling scale of 25%, and the mapping of all samples to the embedded space based on the NN trained on sub-samples. **(F)** net-SNE under the similar experiments as **(E)**.

Another important feature of NeuralEE, owing to the parametric framework, is its generalizability, i.e., ability of mapping newly observed points from the original expression space to the embedded space (Cho et al., 2018). To validate the generalizability, we compare NeuralEE with net-SNE. We first apply and train NeuralEE on a subset of samples (a quarter sample in size). With the trained NN, we then directly map the left-out samples onto the embedded space and compare the visualizations. We test on three scales of sub-sampling using 10%, 25%, and 50% of the original sample size. Figures 3A,B shows the embedding trained on the full dataset with NeuralEE and net-SNE. The top line panels of Figures 3C,D are the visualization of the embeddings based on the sub-samples. The bottom line panels of Figures 3C,D are the mapping of all samples to the embedded space based on the trained NN that corresponds top panels. We see that even when training on 10% of samples and with the subsample visualization shattered into broken clusters, the mapping visualization of NeuralEE (the right column in Figure 3C) is still rather robust and consistent with the result at Figure 3A. In contrast, the mapping visualization of net-SNE is only comparable to its full sample visualization on a 50% scale. Explicit distortions to the true structure are observed with mappings based on 10% and 25% subsample trainings (Figure 3D). We also test the generalizability on real biological data by setting the sub-sampling scale to 25%. On **HEMATO**, **PBMC**, and **RETINA** datasets, both NeuralEE and net-SNE show consistent results of mapping visualization to full sample embedding (Supplementary Figure 5). On the **CORTEX** data, NeuralEE performs well; however, net-SNE has a poor subsample embedding and, thus, an unsatisfactory mapping visualization (Figures 3E,F).

### 3.3. NeuralEE Is Scalable and Efficient

Next, we demonstrate the scalability of NeuralEE, which is another desired property with ever-growing data size nowadays. We apply NeuralEE, UMAP, and FIt-SNE to the **BRAIN-LARGE** dataset. Since there are over 25,000 genes in the **BRAIN-LARGE** dataset, we apply PCA initialization to the data; that is, we retain the top 50 PCs for further analysis. As the data is huge, we will apply NeuralEE-SO instead to reduce memory consumption. In this case, we set the batch size to 5,000 cells.

**Figure 4:**
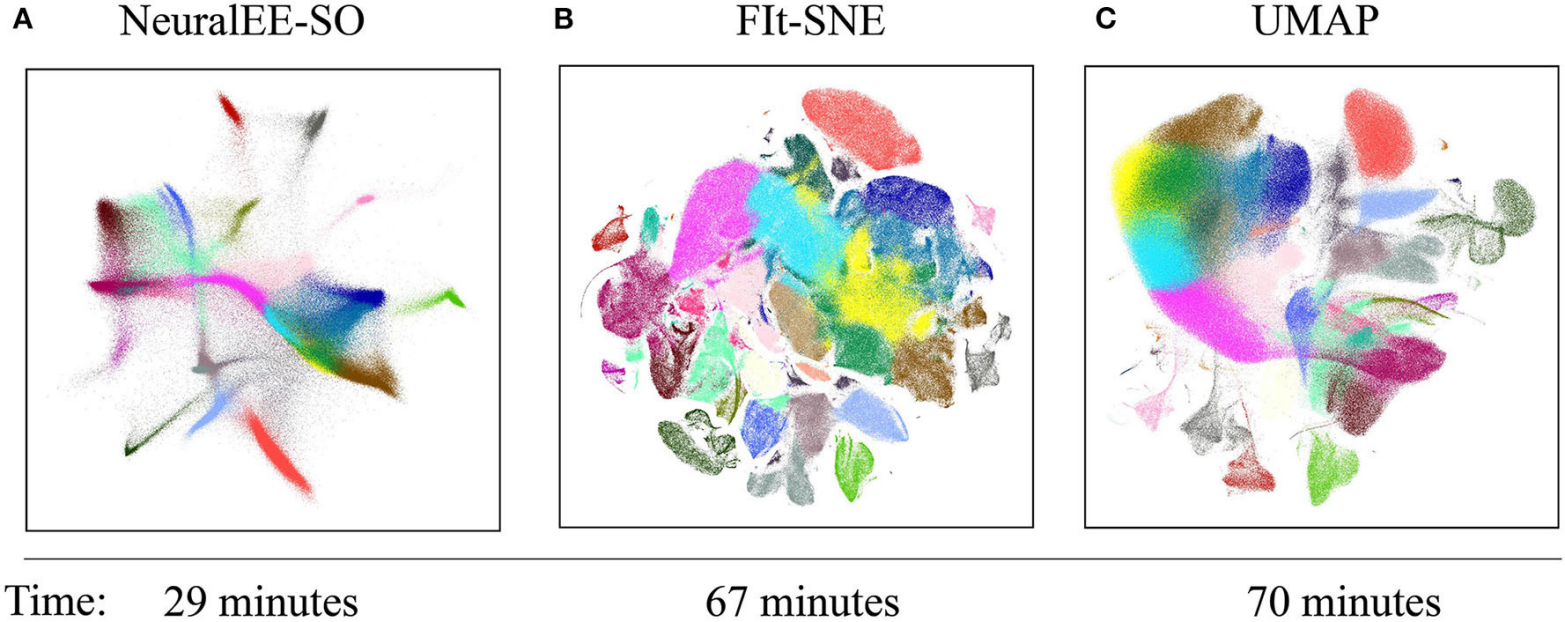
Embedding results on 1.3 million mouse brain cells dataset. **(A)** NeuralEE-SO with batch size as 5,000. **(B)** FIt-SNE. **(C)** UMAP. Labels represent different clusters of Louvain community detection algorithm (Blondel et al., 2008). As NeuralEE belongs to parametric methods, when applied on million-size data, it could return several outliers on the embedded space, we manually delete them to make layout tighter. The raw embedding is showed at Supplementary Figure 7.

Figure 4 shows comparable results among the three methods. The colors are annotated according to Wolf et al. (2018). All methods visualize clusters clearly, with the clusters arranged by NeuralEE-SO in more connected manner, while in more separate and shatter layout manner by FIt-SNE. Besides the visualization, NeuralEE-SO also performs more efficiently, which only takes 29 min to visualize these 1.3 million mice brain cells, and the running times for FIt-SNE and UMAP are 67 and 70 min, respectively.

## 4. Discussion

We develop NeuralEE, a GPU-accelerated dimensionality reduction method for visualization of large-scale scRNA-seq data.

NeuralEE applies a NN framework to parameterize the embedding, where the coordinate of data point in the original space is mapped to the embedded space. The mapping function thus trained allows NeuralEE to be generalizable, where the newly observed data can be directly mapped to the embedded manifold based on its features in the original space. After training NerualEE on a small set of sub-samples, the mapping visualization of remaining samples is comparable to the result of display based on full sample optimization.

We also provide a version of NeuralEE with application of the mini-batch trick. This is especially useful when dealing with large-scale dataset since a significant portion of cells are redundant in large-scale scRNA-seq data as experimentally demonstrated by Cho et al. (2018). In this way, the attractive and repulsive weights in the loss function of NeuralEE are optimized within each random batch which will greatly reduce the memory consumption and make NeuralEE scalable to datasets with millions of samples. Although we show that the batch size has minor effect on the resulting embedding, theoretically however, mini-batch trick applied on NeuralEE cannot guarantee the performance of the embedding, specifically on data with median or small size. As a result, we recommend using the mini-batch trick only when necessary, while in situations with small- or moderate-scale working with NeuraEE instead of NeuralEE-SO might be more robust. We also offer the option of online learning in NeuralEE-SO, where, by forming the newly observed data as new batches, the trained NeuralEE model can be updated.

The deep-learning-based design has been more popularly introduced in dimensionality reduction methods for scRNA-seq data owning to its scalable property. The variational autoencoder proposes a distinct family of generative process where the high-dimensional observed data can be generated from the low-dimensional latent space. For instance, scVI follows Zero-Inflated Negative Binomial for generative process (Risso et al., 2018) and assumes a decomposable form for approximated posterior inference. The posterior distributions parameters of low-dimensional latent variables are inferred by the learned parametric mapping from NN. These methods are powerful and work well when data approximately fits the model assumptions (Wu and Zhang, 2020). NeuralEE, on the other hand, focuses on preserving the intrinsic structure both locally and globally of the data, by simply utilizing the EE objective function and taking the advantage of scalability and parametric property of NN. We believe that such design may extend the capacity of applying EE to larger scale data, well preserving good properties for embedding and visualization.

NeuralEE also takes the advantage of utilizing GPU to accelerate the optimization. The optimization of NeuralEE mainly consists of matrix computation, and it can therefore be dramatically accelerated if its computation can be parallelizable. Although some of these methods leverage the merits of parallel computation, however, the optimizers for t-SNE and most of its variant methods, including EE, are only applicable on CPU. The number of CPU cores on a personal computer or even on a computation server is limited and normally incomparable to that of a GPU chip. By applying a GPU-based code design, NeuralEE can be easily implemented on a workstation or personal computer equipped with a regular NVIDIA GPU chip. In our experiment, it takes only half an hour to visualize 1.3 million mice brain cells on a NVIDIA GeForce GTX 1080 Ti GPU device and 64G computer memory (Memory consumption is illustrated at Supplementary Table 3).

In summary, NeuralEE is a scalable, generalizable, and, more importantly, GPU-accelerated dimensionality reduction method for visualization of scRNA-seq data.

## Data Availability Statement

NeuralEE is freely available at https://github.com/HiBearME/NeuralEE and its corresponding documentation is available at https://neuralee.readthedocs.io. All datasets analyzed in this paper are public. **CORTEX**, **HEMATO**, **PBMC**, **RETINA** and **BRAIN-LARGE** can be referenced at https://github.com/romain-lopez/scVI-reproducibility, and **ArtificialTree** can be referenced at https://github.com/KrishnaswamyLab/PHATE.

## Author Contributions

LM, LW, and FG conceived the NeuralEE models. JX, LM, and LW designed the NeuralEE models and performed the simulation study and real data set analysis. JX programmed the NeuralEE package. LM, LW, and JX wrote the whole manuscript. All authors read and approved the final manuscript.

## Conflict of Interest

The authors declare that the research was conducted in the absence of any commercial or financial relationships that could be construed as a potential conflict of interest.
