# Supplementary material for: NeuralEE: A GPU-Accelerated Elastic Embedding Dimensionality Reduction Method for Visualizing Large-Scale scRNA-Seq Data
Source: Front Genet. 2020 Oct 6;11:786. doi: 10.3389/fgene.2020.00786 (PMC7587292; doi:10.3389/fgene.2020.00786)
Supplement: Supplementary file 1 [file Data_Sheet_1.pdf]

NeuralEE: A GPU-Accelerated Elastic Embedding Dimensionality Reduction Method for Visualizing Large-Scale scRNA-Seq Data

1 SUPPLEMENTARY NOTES

1.1 Pseudocode of NeuralEE

Algorithm S1 NeuralEE

Input: sample-feature matrix Y, perplexity K, trade-off factor λ, batches N_b, the neural network Net_θ with parameters θ and epochs N_e
Output: optimized neural networks parameter θ and low-dimensional embedding X
step 1: randomly partition data and calculate attractive and repulsive weights matrices
$(Y_1, Y_2, \ldots, Y_{N_b}) \leftarrow RandomlyPartition(Y, N_b)$ # N_b can be 1, when not applied with stochastic optimization
for $n \in [1, N_b]$ **do**
    $W_n^- \leftarrow EuclideanDistance(Y_n)$
    $W_n^+ \leftarrow Affinity(Y_n, K)$ # Gaussian affinity[1] or entropic affinity[2]
    $W_n^-, W_n^+ \leftarrow (W_n^- + W_n^{-T})/2, (W_n^+ + W_n^{+T})/2$ # symmetrization
    $ZeroizeDiagonal(W_n^-, W_n^+)$
    $L_n^+ \leftarrow Diagnol(ColumnSum(W_n^+)) - W_n^+$
end for
step 2: optimize parameters of the neural network
initialize θ
for $epoch \in [1, N_e]$ **do**
    for $n \in [1, N_b]$ **do**
        $X_n \leftarrow Net_\theta(Y_n)$
        $\widetilde{W_n^-} \leftarrow W_n^- \circ exp(X_n)$ # $\circ$ means Hadamard product
        $\widetilde{L_n^-} \leftarrow Diagnol(ColumnSum(\widetilde{W_n^-})) - \widetilde{W_n^-}$
        $G_{EE} \leftarrow 4X_n(L_n^+ - \lambda\widetilde{L_n^-})$ # gradient of EE
        $G_\theta \leftarrow BackPropagation(Net_\theta, X_n, G_{EE})$ # gradient from backpropagation[3] by chain rule
        $\theta \leftarrow NonlinearOptimizer(\theta, G_\theta)$ # default: Adam[4]
    end for
end for
step 3: complete embedding by directly mapping
$X = Net_\theta(Y)$

[1] (Hinton and Roweis, 2003), [2] (Vladymyrov and Carreira-Perpinan, 2013), [3] (Lecun et al., 1990), [4] (Kingma and Ba, 2015)

1.2 Details of Data

Details of all biological data refer to (Lopez et al., 2018).

HEMATO. This dataset with continuous gene expression variations from hematopoeitic progenitor cells (Tusi et al., 2018) contains 4,016 cells and 7,397 genes. The library *basal-bm1*, which was of poor quality based on authors recommendation, is removed. Their population balance analysis (Weinreb et al., 2017) result is used as a potential function for differentiation.

CORTEX. The Mouse Cortex Cells dataset from (Amit et al., 2015) contains 3005 mouse cortex cells and gold-standard labels for seven distinct cell types. Each cell type corresponds to a cluster to recover. Top 558 genes are retained, ordered by variance as in (Prabhakaran et al., 2016).

PBMC. scRNA-seq data from two batches of peripheral blood mononuclear cells (PBMCs) from a healthy donor (4K PBMCs and 8K PBMCs) (Zheng et al., 2017) is considered. Quality control metrics is derived using the cellrangerRkit R package (v.1.1.0). Quality metrics are extracted from CellRanger throughout the molecule specific information file. After filtering as in (Cole et al., 2019), 12,039 cells are extracted with 10,310 sampled genes and get biologically meaningful clusters with the software Seurat (Macosko et al., 2015). Then genes that we could not match with the bulk data used for differential expression are filtered to be left with $g = 3346$.

RETINA. The dataset of bipolar cells from (Shekhar et al., 2016) contains 27,499 cells and 13,166 genes coming from two batches after their original pipeline for filtering. Cluster annotation from 15 cell-types from the author is used.

BRAIN-LARGE. This dataset consists of 1.3 million mouse brain cells, spanning the cortex, hippocampus and subventricular zone, and is profiled with 10x chromium (10x Genomics, 2017). The raw gene expression count matrix includes 1,306,127 cells and 27,998 genes.

The artificial tree data shown in Figure 2A is constructed as (Moon et al., 2019).

ArtificialTree. The first branch consists of 100 linearly spaced points that progress in the first four dimensions. All other dimensions were set to zero. The 100 points in the second branch are constant in the first four dimensions with a constant value equal to the end point of the first branch. The next four dimensions then progress linearly in this branch while all other dimensions were set to zero. The third branch is constructed similarly except the progression occurs in dimensions 9–12 instead of dimensions 5–8. All remaining branches are constructed similarly with some variation in the length of the branches. At each end point and branch point, 40 points are added 40 and zero mean Gaussian noise with a $s.d.$ of 7 is added. This construction models a system where progression along a branch corresponds to an increase in gene expression in several genes. Additional noise dimensions are also added, bringing the total dimensionality of the data to 60.

1.3 The specific structure of Neural Network

The default NN structure of our NeuralEE is designed as follows: It has two hidden layers with 50 nodes, and both of them are equipped with Batch Normalization (Ioffe and Szegedy, 2015) and ReLU activation function. The final output layer, which is the embedding layer in our context, is without any activation function. We apply this structure to **CORTEX**, **HEMATO**, **PBMC**, **RETINA** and **BRAIN-LARGE**. As for **ArtificialTree**, since the data size is relatively small and the architecture is rather distinct, we instead two hidden layers with 32 nodes and 8 nodes of default setting.

REFERENCES

10x Genomics (2017). Support: single cell gene expression datasets. *10x Genomics*

Amit, Z., MuñOz-Manchado, A. B., Simone, C., Peter, L., Gioele, L. M., Anna, J., et al. (2015). Brain structure. cell types in the mouse cortex and hippocampus revealed by single-cell rna-seq. *Science* 347, 1138–42

Cole, M. B., Risso, D., Wagner, A., DeTomaso, D., Ngai, J., Purdom, E., et al. (2019). Performance assessment and selection of normalization procedures for single-cell rna-seq. *Cell* 8, 315–328

Hinton, G. and Roweis, S. (2003). Stochastic neighbor embedding. *Advances in Neural Information Processing Systems* 15, 833–840

Ioffe, S. and Szegedy, C. (2015). Batch Normalization: Accelerating Deep Network Training by Reducing Internal Covariate Shift. *arXiv e-prints* , arXiv:1502.03167

Kingma, D. P. and Ba, J. (2015). Adam: A method for stochastic optimization. *International Conference on Learning Representations*

Lecun, Y., Boser, B. E., Denker, J. S., Henderson, D., Howard, R. E., Hubbard, W., et al. (1990). Handwritten digit recognition with a back-propagation network. *Advances in Neural Information Processing Systems* 2, 396–404

Lopez, R., Regier, J., Cole, M. B., Jordan, M. I., and Yosef, N. (2018). Deep generative modeling for single-cell transcriptomics. *Nature Methods* 15, 1053–1058

Macosko, E., Basu, A., Satija, R., Nemesh, J., Shekhar, K., Goldman, M., et al. (2015). Highly parallel genome-wide expression profiling of individual cells using nanoliter droplets. *Cell* 161, 1202–1214

Moon, K. R., van Dijk, D., Wang, Z., Gigante, S., Burkhardt, D. B., Chen, W. S., et al. (2019). Visualizing structure and transitions in high-dimensional biological data. *Nature Biotechnology* 37, 1482–1492

Prabhakaran, S., Azizi, E., Carr, A., and Pe'Er, D. (2016). Dirichlet process mixture model for correcting technical variation in single-cell gene expression data. In *International Conference on International Conference on Machine Learning*

Shekhar, K., Lapan, S. W., Whitney, I. E., Tran, N. M., Macosko, E. Z., Kowalczyk, M., et al. (2016). Comprehensive classification of retinal bipolar neurons by single-cell transcriptomics. *Cell* 166, 1308–1323

Tusi, B. K., Wolock, S. L., Weinreb, C., Hwang, Y., Hidalgo, D., Zilionis, R., et al. (2018). Population snapshots predict early haematopoietic and erythroid hierarchies. *Nature* 555, 54–60

Vladymyrov, M. and Carreira-Perpinan, M. A. (2013). Entropic affinities: properties and efficient numerical computation. *International Conference on Machine Learning* , 477–485

Weinreb, C., Wolock, S., Tusi, B. K., Socolovsky, M., and Klein, A. M. (2017). Fundamental limits on dynamic inference from single-cell snapshots. *Proc Natl Acad Sci USA* 115, E2467

Zheng, G. X. Y., Terry, J. M., Belgrader, P., Ryvkin, P., Bent, Z. W., Wilson, R., et al. (2017). Massively parallel digital transcriptional profiling of single cells. *Nature Communications* 8, 14049

2 SUPPLEMENTARY TABLES AND FIGURES

2.1 Tables

Table S1. Generalization error of different dimensionality reduction methods on the **ArtificialTree** data. Based on the optimal K-nearest neighbor classifier on the low-dimensional space, the minimum cross validation errors of each methods are chosen as the corresponding generalization errors.

Methods	NeuralEE	NeuralEE-SO	EE	tSNE	UMAP	PHATE	PCA
Generalization error	0.0792	0.0848	0.0792	0.0917	0.0979	0.0993	0.3028
Optimal K	9	4	4	15/16	9	9	29

Table S2. Hyperparameters selection. In most cases, default hyperparameters of corresponding algorithms are selected, while in the case of Fit-SNE on **BRAIN-LARGE**, the selected hyperparameters follow the code of original paper, and in the case of net-SNE, the number of layers is set as 3, since if the number of layers is set as default (2), it would be poor to approximate the mapping function in some cases (not show). In the case of NeuralEE-SO on large-scale **BRAIN-LARGE**, batch size is set as 5000.

**Algorithms**	**Hyperparameters**		
	**ArtificialTree**	**HEMATO CORTEX PBMC RETINA**	**BRAIN-LARGE**
NeuralEE	$size = 1.0$<br>$lambda = 1$<br>$perplexity = 30$	$size = 1.0$<br>$lambda = 1$<br>$perplexity = 30$	
NeuralEE-SO	$size = 0.25$<br>$lambda = 1$<br>$perplexity = 30$	$size = 0.25$<br>$lambda = 1$<br>$perplexity = 30$	$size = 5000$<br>$lambda = 1$<br>$perplexity = 30$
EE	$lambda = 1$<br>$perplexity = 30$	$lambda = 1$<br>$perplexity = 30$	
t-SNE	$perplexity = 30$	$perplexity = 30$	
FIt-SNE			$perplexity = 30$<br>$max\_iter = 4000$<br>$stop\_early\_exag\_iter = 2000$
net-SNE	$perplexity = 30$<br>$num\text{-}layers = 3$	$perplexity = 30$<br>$num\text{-}layers = 3$	
UMAP	$n\_neighbors = 15$<br>$min\_dist = 0.5$	$n\_neighbors = 15$<br>$min\_dist = 0.5$	$n\_neighbors = 15$<br>$min\_dist = 0.5$
PHATE	$a = 40$<br>$k = 5$	$a = 40$<br>$k = 5$	

Table S3. Approximated memory consumption for some cases. It's mainly allocated for the multiple batches of attractive and repulsive matrices, approximated by $\mathrm{DataSize} \times \mathrm{BatchSize} \times 4 \times 2/1024^3\,\mathrm{(GBytes)}$. Lower batch size of stochastic optimization will enable applications on computers with limited memory.

DataSize	BatchSize	Memory consumption
10,000	10,000	0.745GB(PC)
100,000	10,000	7.45GB(PC)
1,000,000	10,000	74.5GB(WorkStation)
1,000,000	1,000	7.45GB(PC)
1,300,000	5,000	48.4GB(WorkStation)

2.2 Figures

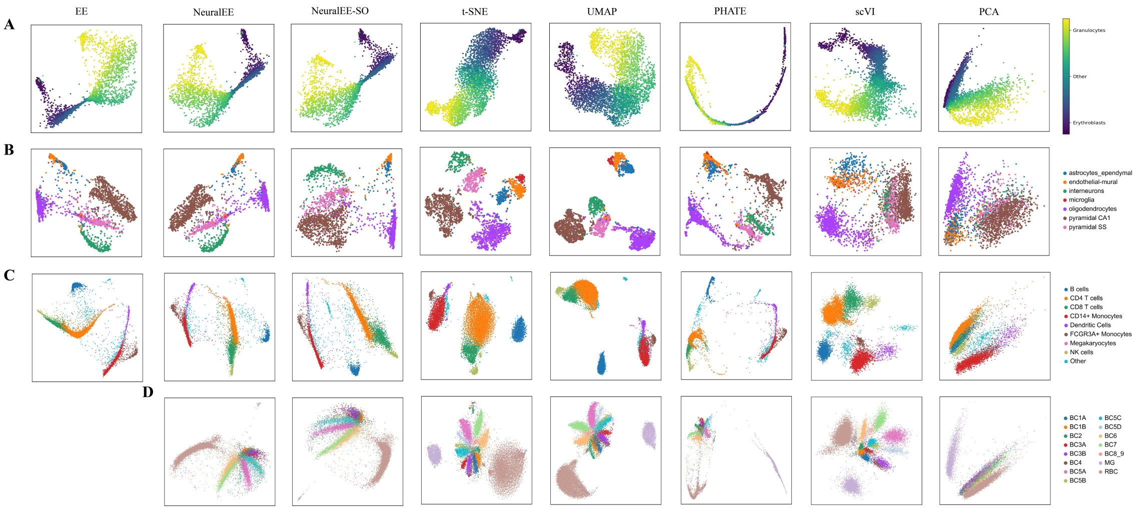

Figure S1. Comparison of NeuralEE to other visualization methods, EE, t-SNE, UMAP, PHATE, scVI and PCA, on **(A) HEMATO** data, **(B) CORTEX** data, **(C) PBMC** data and **(D) RETINA** data.

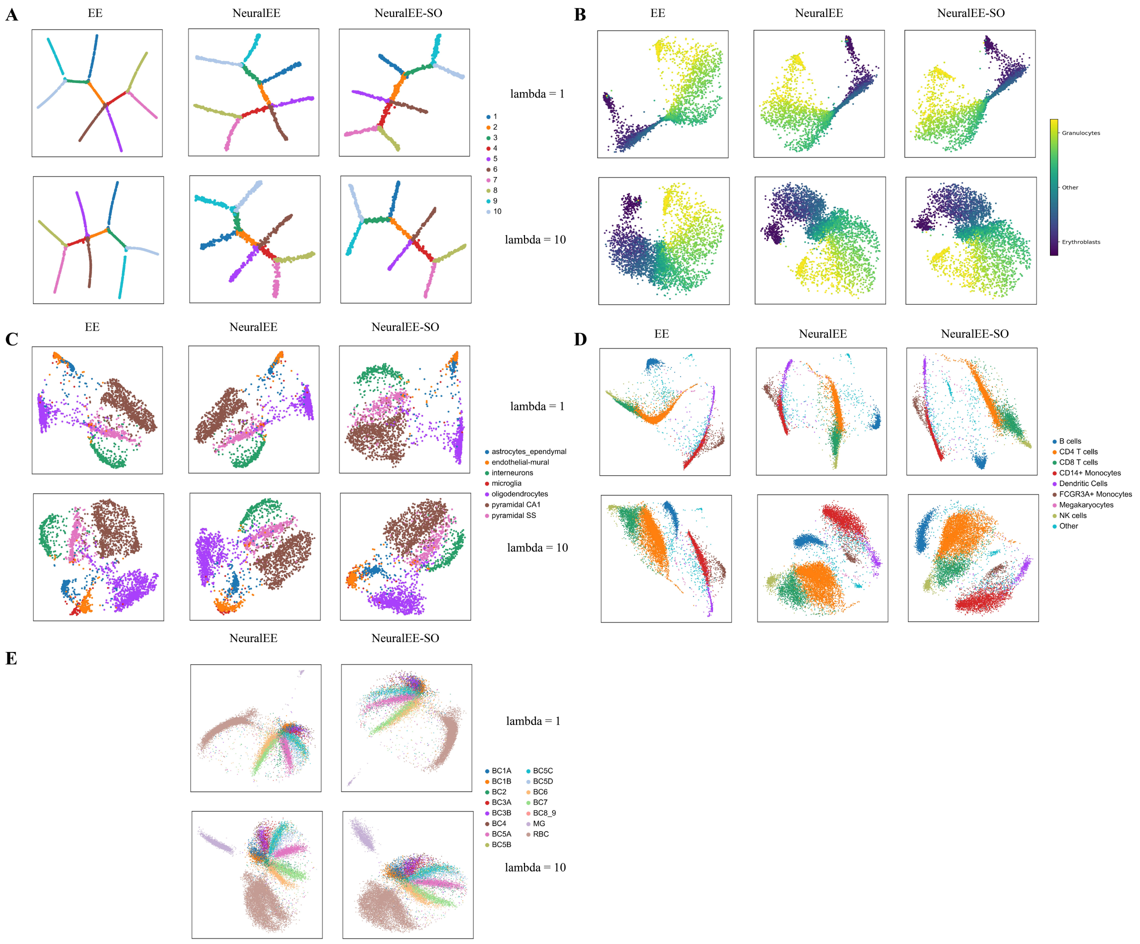

Figure S2. Influence of different trade-off coefficient parameters ($\lambda = 1, 10$) on embedding. On **(A) ArtificialTree** data, there is not significant difference between the two cases. And on **(B) HEMATO** data, **(C) CORTEX** data, **(D) PBMC** data, **(E) RETINA** data, there is a little difference between the local structure of two layouts, however the global structure maintains as the same. Furthermore, the layout of $\lambda = 10$ presents more distributed than that of $\lambda = 1$ on the local structure.

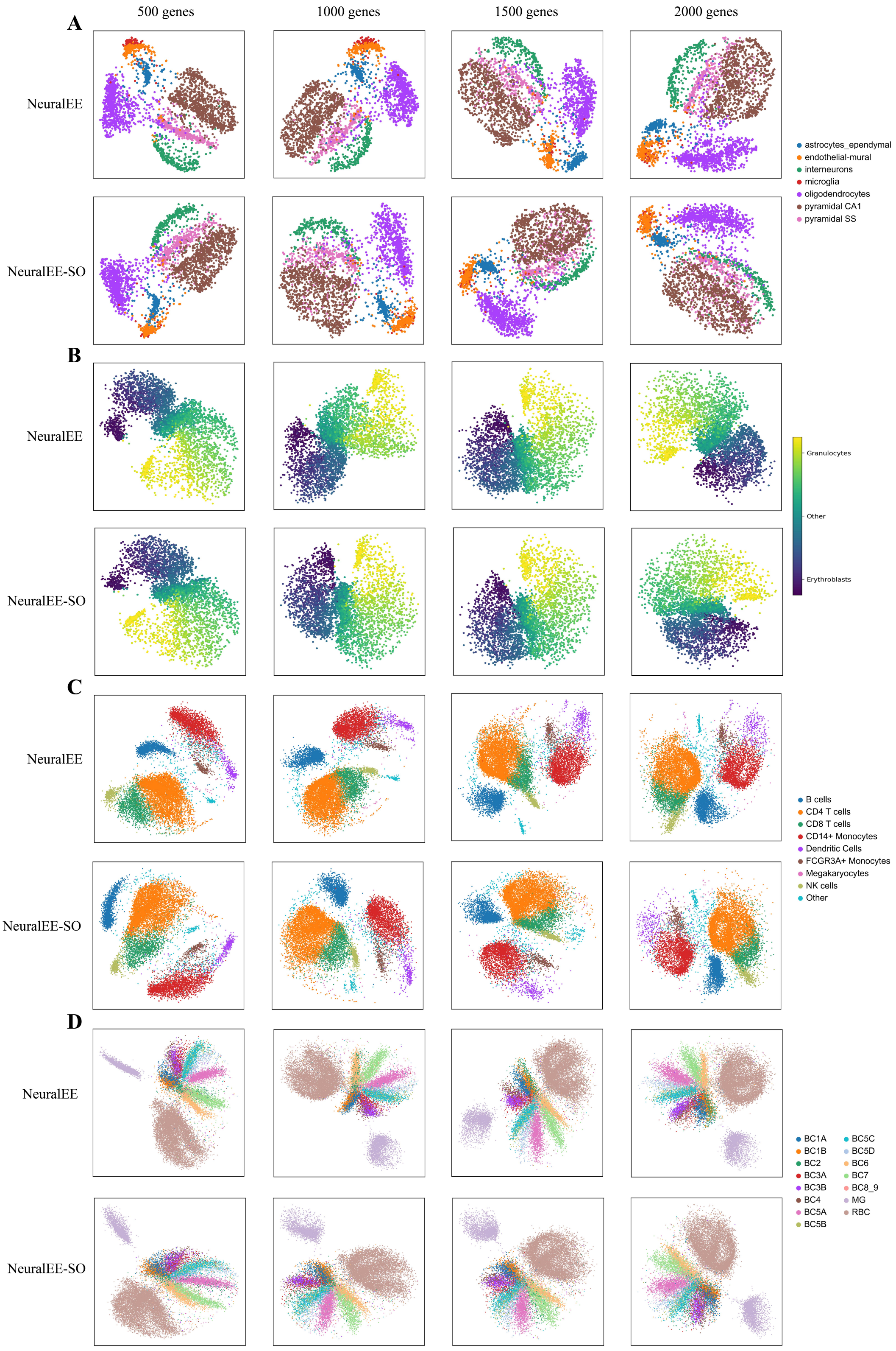

Figure S3. NeuralEE with the setting of different numbers of top gene retained. Exclude genes with low expression variance, and retain the top genes. **(A) CORTEX** data. **(B) HEMATO** data. **(C) PBMC** data. **(D) RETINA** data. To achieve more details on local structure of embedding for comparison, we set λ = 10, making layout more distributed. The structure maintains as the same among the setting of different numbers of top gene retained.

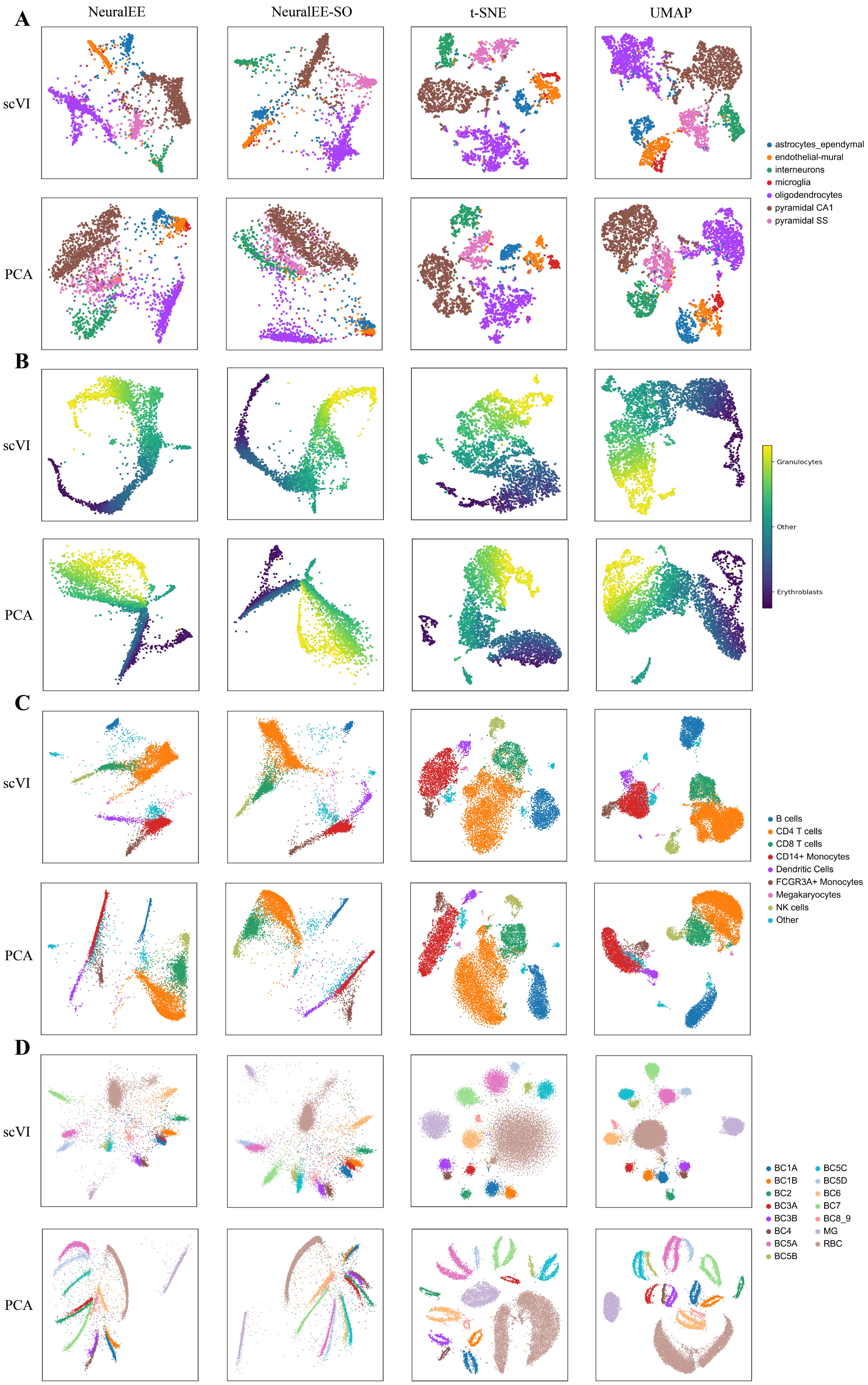

Figure S4. Embedding results based on 50 PCs or latent variables learned by scVI. **(A) CORTEX** data. **(B) HEMATO** data. **(C) PBMC** data. **(D) RETINA** data.

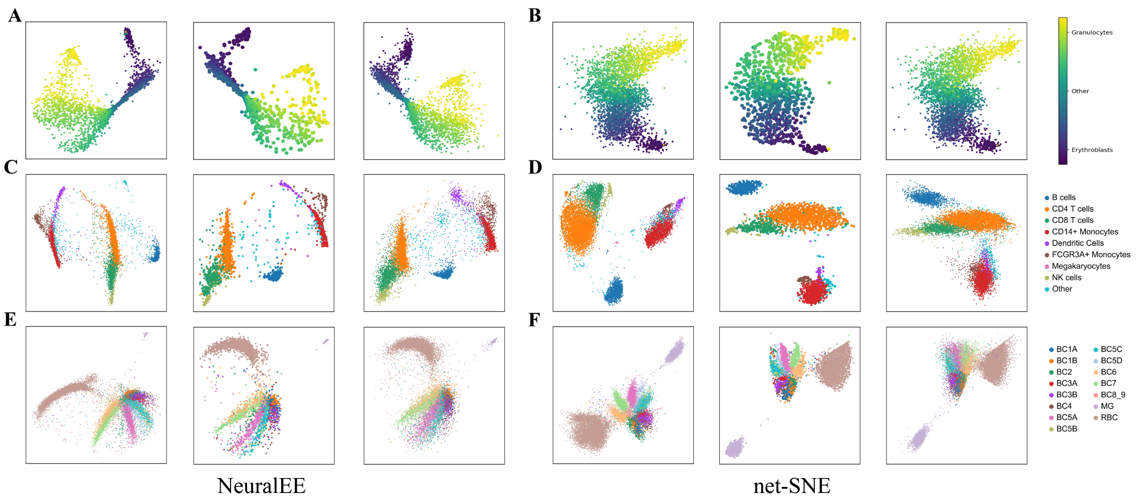

Figure S5. **(A)** From left to right is, NeuralEE on the entire **HEMATO** data, NeuralEE based on the sub-samples with sub-sampling scale as 25% and the mapping of all samples to the embedded space based on the NN trained on sub-samples. **(B)** net-SNE under the similar experiments as **(A)**. **(C)** NeuralEE and **(D)** net-SNE under the similar experiments as **(A)** on **PBMC** data. **(E)** NeuralEE and **(F)** net-SNE under the similar experiments as **(A)** on **RETINA** data.

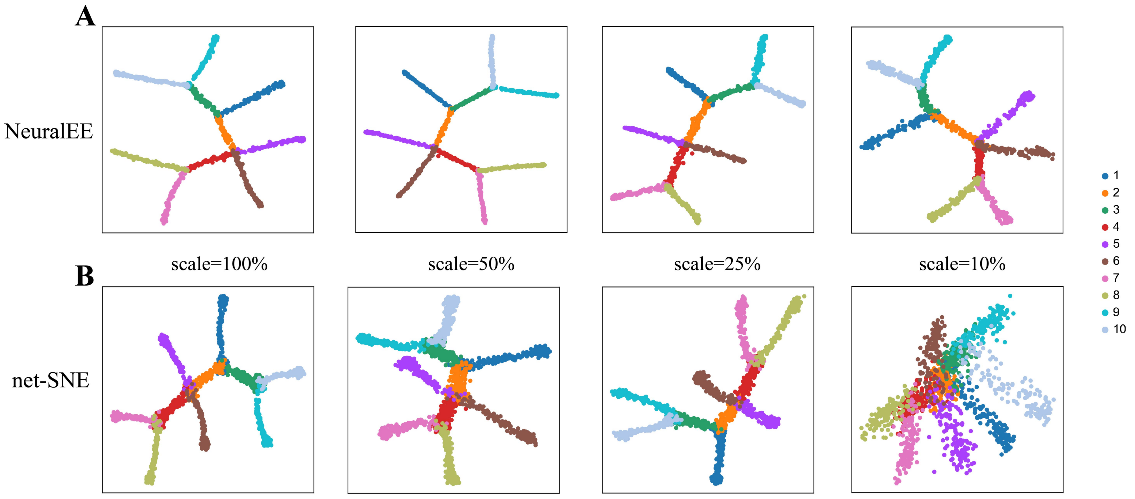

Figure S6. **(A)** From left to right is, NeuralEE-SO on **ArtificialTree** data with batch scale as 100%, 50%, 25% and 10% respectively. NeuralEE-SO with batch scale as 100% actually is NeuralEE without stochastic optimization. **(B)** net-SNE under the similar experiments as **(A)**.

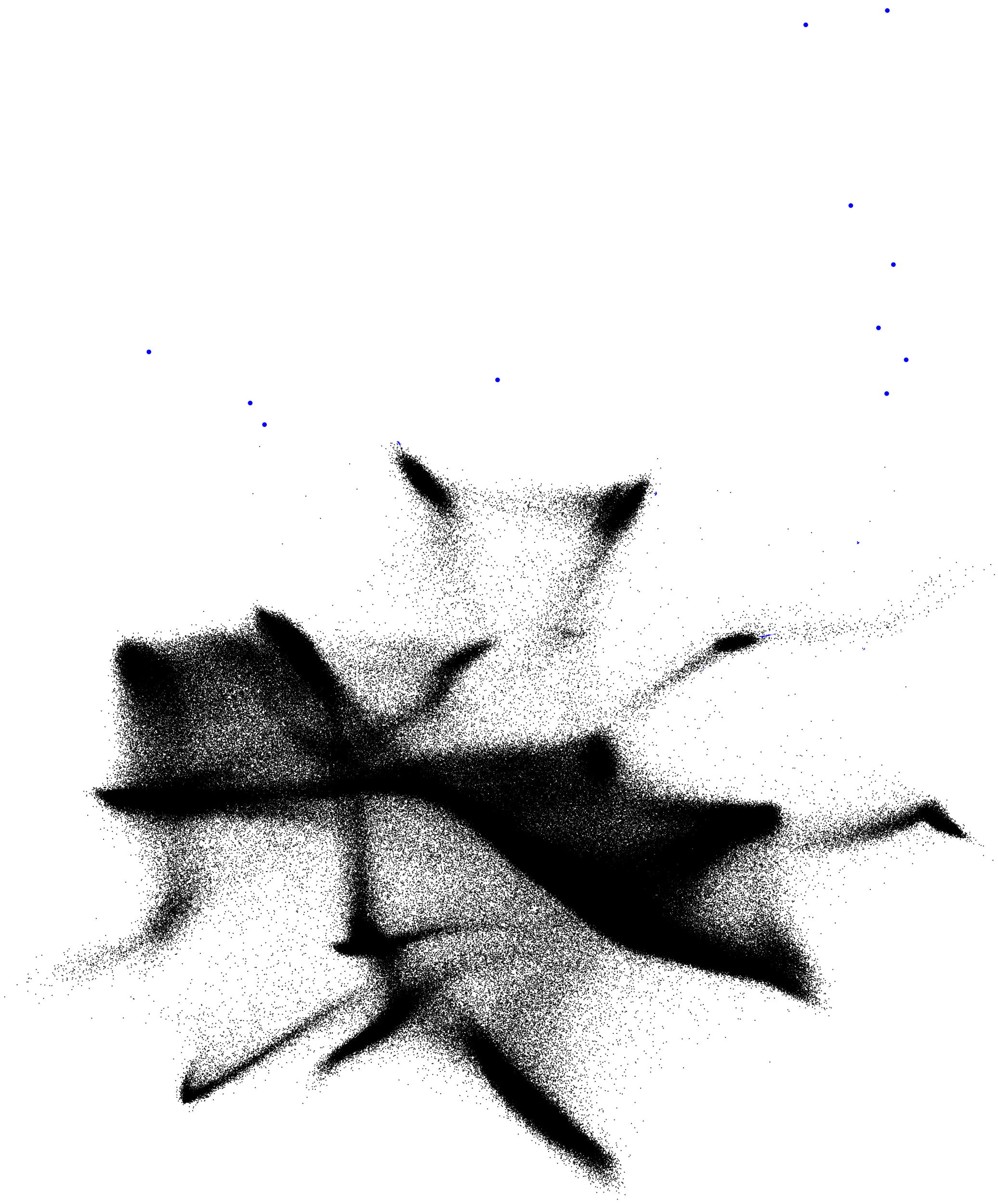

Figure S7. The entire NeuralEE embedding on **BRIAN-LARGE**. Blue and enlarged dots are which we manually delete to make layout tighter.
